# β-D-Glucan promotes NF-κB activation and ameliorates high-LET carbon-ion irradiation-induced human umbilical vein endothelial cell injury

**DOI:** 10.55730/1300-0144.5731

**Published:** 2023-09-21

**Authors:** Fang LIU, Yanting WEI, Zhuanzi WANG

**Affiliations:** 1 International Genome Center, Jiangsu University, Zhenjiang, Jiangsu, P.R. China; 2 Institute of Modern Physics, Chinese Academy of Sciences, Lanzhou, Gansu, P.R. China

**Keywords:** *Saccharomyces cerevisiae*-derived-β-D-glucan, human umbilical vein endothelial cells, high-LET carbon-ion irradiation, radioprotection, NF-κB, prosurvival-related gene expression

## Abstract

**Background/aim:**

Heavy-ion irradiation seriously perturbs cellular homeostasis and thus damages cells. Vascular endothelial cells (ECs) play an important role in the pathological process of radiation damage. Protecting ECs from heavy-ion radiation is of great significance in the radioprotection of normal tissues. In this study, the radioprotective effect of β-D-glucan (BG) derived from *Saccharomyces cerevisiae* on human umbilical vein endothelial cell (EA.hy926) cytotoxicity produced by carbon-ion irradiation was examined and the probable mechanism was established.

**Materials and methods:**

EA.hy926 cells were divided into seven groups: a control group; 1, 2, or 4 Gy radiation; and 10 μg/mL BG pretreatment for 24 h before 1, 2, or 4 Gy irradiation. Cell survival was assessed by colony formation assay. Cell cycles, apoptosis, DNA damage, and reactive oxygen species (ROS) levels were measured through flow cytometry. The level of malondialdehyde and antioxidant enzyme activities were analyzed using assay kits. The activation of NF-κB was analyzed using western blotting and a transcription factor assay kit. The expression of downstream target genes was detected by western blotting.

**Results:**

BG pretreatment significantly increased the survival of irradiated cells, improved cell cycle progression, and decreased DNA damage and apoptosis. The levels of ROS and malondialdehyde were also decreased by BG. Further study indicated that BG increased the antioxidant enzyme activities, activated Src, and promoted NF-κB activation, especially for the p65, p50, and RelB subunits. The activated NF-κB upregulated the expression of antioxidant protein MnSOD, DNA damage-response and repair-related proteins BRCA2 and Hsp90α, and antiapoptotic protein Bcl-2.

**Conclusion:**

Our results demonstrated that BG protects EA.hy926 cells from high linear-energy-transfer carbon-ion irradiation damage through the upregulation of prosurvival signaling triggered by the interaction of BG with its receptor. This confirms that BG is a promising radioprotective agent for heavy-ion exposure.

## 1. Introduction

Radiotherapy is an essential tool for cancer treatment. Its main goal is to efficiently eradicate tumors while sparing surrounding healthy tissues. One of the recent developments in radiotherapy modalities is high linear-energy-transfer (LET) heavy-ion therapy [1]. High-LET heavy-ion therapy is of particular interest because of its improved depth dose distribution and dense ionizing track in comparison to low-LET rays (X/γ-rays); its better physical dose distribution makes it possible to irradiate tumors with greater precision while minimizing the damage to surrounding healthy tissues, and dense ionizing radiation induces more damage per unit of dose, more severe or even permanent cell cycle arrest, and highly lethal effects even on radioresistant tumors [1,2]. Normal tissue damage due to therapeutic or accidental radiation exposure is a pervasive threat. With increased use of heavy-ion radiotherapy, developing effective preventive agents becomes an important goal for the protection of healthy tissues from heavy-ion irradiation damage.

The vast network of microcapillaries and other vessels in human tissues makes it a major target of radiation damage. Endothelial cells (ECs), forming the inner layer of all vascular structures, are especially sensitive to irradiation. X-rays of 0.05 Gy could induce DNA double-strand breaks in ECs [3]. High-LET heavy-ion irradiation can be more damaging for ECs than low-LET irradiation [4,5]. Studies also indicated time- and radiation quality-dependent changes of the EC response to irradiation, and the irradiation impact was shown to be more pronounced and longer lasting for heavy-ion radiation than for photons [6]. ECs play an important role in the pathological process of radiation damage. Thus, protecting ECs from heavy-ion radiation damage is of great significance for the radioprotection of normal tissues.

β-Glucan, a natural polysaccharide that can be extracted from fungi, yeast, some bacteria, and cereals [7], is one of the potential radioprotectors. It can scavenge free radicals [8], prevent oxidative damage induced by reactive oxygen species (ROS) [9], mitigate low-LET irradiation-induced DNA damage, and increase the survival of irradiated mice or cells [10–12]. However, the precise radioprotective mechanisms of β-glucan remain unknown, and few studies of the protective effects of β-glucan against high-LET heavy-ion irradiation have been carried out.

β-Glucan is not expressed in mammalian cells, but it can be recognized in mammalian cells by pattern recognition receptors, such as complement receptor-3, dectin-1, lactosylceramides, and scavenger receptors [13]. In the present study, the human umbilical endothelial cell line EA.hy926, which expresses the dectin-1 receptor [14], was used as a model system to investigate the protective effect of β-D-glucan (BG) derived from *Saccharomyces cerevisiae* on the damage induced by high-LET carbon-ion irradiation. The signaling triggered by the interaction of BG with its receptor was studied to elucidate the possible mechanism of BG in exerting its radioprotective effect. To the best of our knowledge, the present study is the first to investigate the effect of BG against high-LET carbon ion-induced damage. The findings will contribute to the development of BG as a potential radioprotective agent for both medical and nonmedical high-LET heavy-ion exposure.

## 2. Materials and methods

### 2.1. Reagents

High-glucose DMEM, fetal calf serum, and penicillin/streptomycin were purchased from HyClone (Logan, UT, USA) while 1X PBS, 1X TBS, and Tween-20 were purchased from Solarbio (Beijing, China). The cell cycle kit, ROS assay kit, malondialdehyde (MDA) assay kit, superoxide dismutase (SOD) assay kit, glutathione peroxidase (GPx) assay kit, catalase (CAT) assay kit, RIPA lysis buffer, phenylmethylsulfonyl fluoride (PMSF), and BCA protein assay kit were purchased from Beyotime Biotechnology (Jiangsu, China). The apoptosis kit and nonfat milk were purchased from BD Biosciences (San Diego, CA, USA). The TransAM NF-κB family transcription factor assay kit and nuclear extract kit were purchased from Active Motif (Carlsbad, CA, USA). Polyethylene difluoride membranes and ECL western blotting detection reagents were purchased from GE Healthcare (Zurich, Switzerland). Anti-γH2AX (Ser-139) mouse monoclonal antibody (ab26350) and secondary Alexa-488 conjugated goat antimouse IgG (ab150113) were purchased from Abcam (Cambridge, MA, USA). Antibodies against p-Src (Tyr416) (6943S), Src (13198S), MnSOD (13141S), BRCA2 (10741S), Hsp90α (8165S), Bax (5023S), Bcl-2 (15071S), β-actin (3700S), HRP-conjugated goat antimouse secondary antibody (7076S), and HRP-conjugated goat antirabbit secondary antibody (7074S) were purchased from Cell Signaling (Danvers, MA, USA).

The human EA.hy926 endothelial cell line [15,16] was obtained from the Cell Resource Center of Shanghai Institutes for Biological Sciences, Chinese Academy of Sciences (Shanghai, China). *S. cerevisiae* BG was provided by the Institute of Modern Physics, Chinese Academy of Sciences (Lanzhou, China).

### 2.2. Cell lines and treatment

Human EA.hy926 endothelial cells were cultured in DMEM supplemented with 10% fetal calf serum and penicillin/streptomycin and incubated at 37 °C in an incubator containing 5% CO_2_. Exponentially growing cells were divided into seven groups: a control group (untreated); irradiation (IR) groups receiving 1, 2, or 4 Gy irradiation; and BG + IR groups receiving 10 μg/mL BG pretreatment for 24 h before 1, 2, or 4 Gy irradiation.

Carbon-ion irradiation was supplied by the Heavy Ion Research Facility in Lanzhou (HIRFL) of the Institute of Modern Physics, Chinese Academy of Sciences. The energy of the carbon ions upon entering the cells was calculated to be 80 MeV/u, corresponding to linear energy transfer (LET) of 31.6 KeV/μm in water, and the dose rate was adjusted to be about 1 Gy/min.

### 2.3. Colony formation assay

Cell survival was assessed by colony formation assay. After carbon-ion irradiation, cells in each group were trypsinized and suspended in complete cell culture medium. An appropriate number of cells were seeded in each 60-mm dish. After 14 days of incubation after irradiation, cells were washed with PBS, fixed with methanol supplemented with 10% acetic acid for 30 min, and stained with 0.5% crystal violet. Colonies with more than 50 remaining cells were counted. The clonogenic survival rate was calculated as follows: (N1/n1) /(N2/n2) ×100%, where N1 is the number of cells forming colonies after irradiation, n1 is the number of seeded cells after irradiation, N2 is the number of cells forming colonies in the control group, and n2 is the number of seeded cells in the control group.

### 2.4. DNA damage and cell cycle distribution measurement

Phosphorylated H2AX (γH2AX) was measured to assess DNA damage. At 2, 24, and 36 h after carbon-ion irradiation, cells were trypsinized and collected by centrifugation. Subsequently, 1 × 10^6^ cells per sample were treated with the transcription factor staining buffer kit and then treated cells were incubated with the anti-γH2AX antibody (Ser-139) overnight at 4 °C. Samples were then washed and resuspended for 1 h in Alexa-488 conjugated secondary antibody. After a second rinsing, cells were resuspended in PBS and analyzed using a FlowSight cytometer (Amnis, Merck Millipore, Burlington, MA, USA), and the images were analyzed using ImageStream Data Exploration and Analysis Software (IDEAS, Merck Millipore) and the mean fluorescence intensity (MFI) was calculated. For each sample, 20,000 cells were considered.

Ethanol-fixed samples were rehydrated, washed twice with PBS, and treated with the cell cycle kit according to the manufacturer’s instructions. Cell cycle distribution was then analyzed with FlowJo 7.6.1 software (Tree Star, Portland, OR, USA) using the original histogram of DNA content as measured with the FlowSight cytometer. For each sample, 20,000 cells were considered.

### 2.5. Apoptosis measurement

Cells were collected, washed twice with PBS, and treated with the apoptosis kit according to the manufacturer’s instruction. Af﻿﻿ter being double-stained with annexin V-FITC and PI, cells were examined with the FlowSight cytometer and apoptosis was analyzed using IDEAS. For each sample, 20,000 cells were considered.

### 2.6. Assessment of intracellular ROS

ROS levels were analyzed by measuring the mean fluorescence intensity (MFI) of 2’,7’-dichlorofluorescein (DCF). In this process, 1 × 10^6^ cells were incubated with 10 μM DCF-DA for 20 min at 37 °C. After that, cells were washed three times with PBS and collected using the FlowSight cytometer. Data were analyzed using FlowJo 7.6.1 software.

### 2.7. Assessment of MDA and enzyme activities of SOD, GPx, and CAT

The level of lipid peroxidation as reflected by MDA was analyzed using the MDA assay kit. The levels of SOD, GPx, and CAT enzyme activities in cells were analyzed using the SOD assay kit, GPx assay kit, and CAT assay kit, respectively, following the manufacturer’s instructions.

### 2.8. Assessment of NF-κB

The levels of activation (DNA binding) for NF-κB transcription subunits p65, p50, p52, c-Rel, and RelB in cell nuclear extracts were detected using the highly sensitive TransAM NF-κB family transcription factor assay kit according to the manufacturer’s instructions. This method is based on the enzyme-linked immunosorbent assay (ELISA), and when nuclear extract is added to the 96-well plates, the activated transcription factor binds the oligonucleotide at the consensus binding site and is quantified using the linked antibody, which is specific for the active form of the transcription factor being studied. Nuclear extracts were prepared using the nuclear extract kit.

### 2.9. Western blotting analysis

The expression of pathway-related proteins was detected by western blotting. In this process, 5 × 10^6^ cells were lysed in RIPA lysis buffer with 100 μg/mL phenylmethylsulfonyl fluoride (PMSF) for 30 min on ice and centrifuged at 14,000 × *g* for 10 min at 4 °C. The supernatant was collected and the concentration of protein was estimated using the BCA protein assay kit. Proteins were resolved by 4%–20% SDS-polyacrylamide gel electrophoresis and transferred to polyethylene difluoride membranes. Membranes were blocked with 5% nonfat milk in TBS containing 0.1% Tween-20 (TBST) for 1 h at room temperature. The membranes were then incubated in primary antibody at 4 °C overnight with gentle shaking. p-Src (Tyr416), Src, MnSOD, BRCA2, Hsp90α, Bax, Bcl-2, and β-actin antibodies were used. Afterwards, the membranes were washed three times and incubated with HRP-conjugated secondary antibody. The specific protein bands were detected using ECL western blotting detection reagents and band densities were quantified using ImageJ software (NIH, Bethesda, MD, USA).

### 2.10. Statistical analysis

The data were presented as mean ± standard error based on three independent replications (n = 3). All statistical analysis was performed using Origin software (version 7.0). Statistical significance was determined using Student’s t-test and one-way ANOVA. Differences were considered significant at p < 0.05.

## 3. Results

### 3.1. BG pretreatment enhanced carbon-ion irradiated cell survival

**Figure 1:**
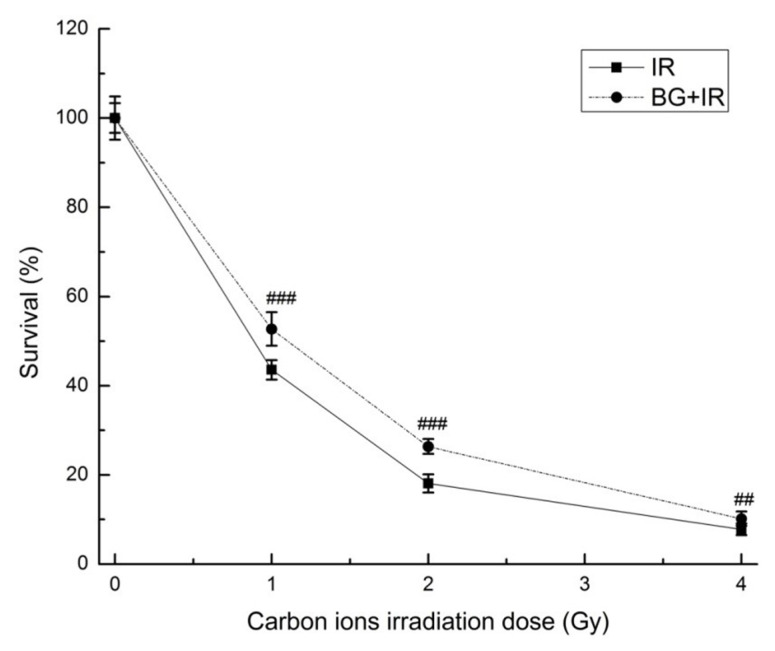
Effect of BG on cell survival. EA.hy926 cells were treated with or without 10 μg/mL BG for 24 h before 0, 1, 2, or 4 Gy carbon-ion irradiation. The clonogenic survival rate of cells was established by colony formation assay. Data are presented as mean ± SE from three independent experiments. ^###^: p < 0.001, ^##^: p < 0.01 versus IR group.

The effect of BG pretreatment on the clonogenic survival of EA.hy926 cells after carbon-ion irradiation was evaluated using the colony formation assay. As shown in Figure 1, the clonogenic survival rate of EA.hy926 cells decreased with increasing radiation dose, and the clonogenic survival rate of cells pretreated with BG before IR increased significantly compared to the clonogenic survival rate of cells irradiated only. The dose-reduction factor (DRF) of BG was calculated as follows: DRF = clonogenic survival rate with radioprotector at specific radiation dose/clonogenic survival rate without radioprotector at specific radiation dose [17]. The DRF was found to be 1.21 at 1 Gy, 1.46 at 2 Gy, and 1.31 at 4 Gy, respectively. These data demonstrated that BG pretreatment could promote the clonogenic survival of EA.hy926 cells and suggested that BG could protect EA.hy926 cells from carbon-ion radiation damage.

### 3.2. BG pretreatment ameliorated cell DNA damage, cell apoptosis, and cell cycle arrest after carbon-ion irradiation

**Figure 2:**
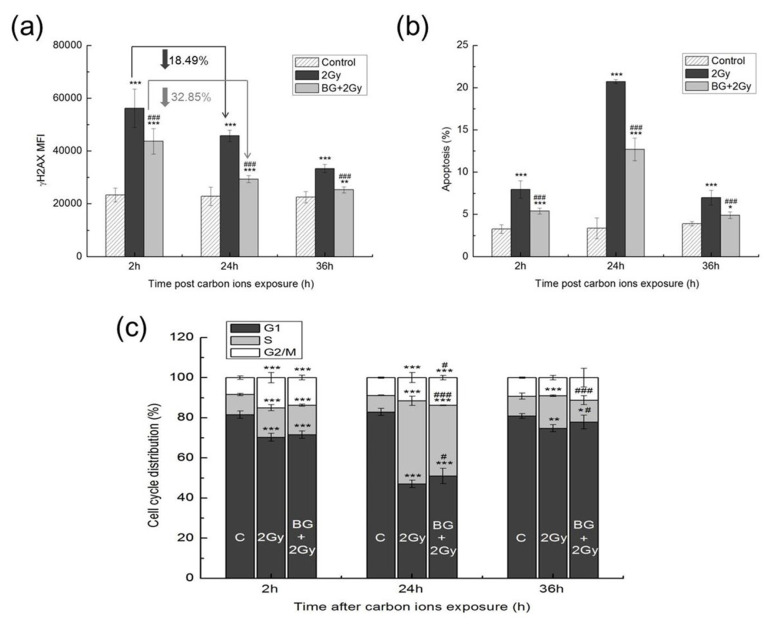
Effect of BG pretreatment on cell damage after 2 Gy carbon-ion irradiation. (a) DNA damage was detected by measuring γH2AX. The γH2AX MFI reduction of each group at 24 h is shown [(“γH2AX MFI at 2 h” – “γH2AX MFI at 24 h”) /“γH2AX MFI at 2h” × 100%]. (b) Percentages of cell apoptosis. (c) Percentages of cell cycle distribution. Data are presented as mean ± SE from three independent experiments. ^***^: p < 0.001, ^**^: p < 0.01, ^*^: p < 0.05 versus control group; ^###^: p < 0.001, ^#^: p < 0.05 versus IR group.

DNA damages, and especially DNA double-strand breaks (DSBs), are closely related to radiation-induced cell death. As BG showed significant radioprotection for cell survival, we evaluated the effect of BG pretreatment on DNA DSBs induced by carbon-ion irradiation. γH2AX was detected by flow cytometry to assess DNA DSBs. The results showed that γH2AX gradually disappeared with incubation time after IR, but even at 36 h after IR, the γH2AX MFI values of the 2 Gy and BG + 2 Gy groups were still significantly higher than those of the control group, which indicated that DNA DSBs in both the 2 Gy and BG + 2 Gy groups were still very serious at 36 h after carbon-ion irradiation (Figure 2a). Compared with the group that received 2 Gy, the γH2AX MFI value of the BG + 2 Gy group was significantly decreased (p < 0.001) at the same time point after IR, which suggested that BG significantly decreased the IR-induced DNA damage of EA.hy926 cells (Figure 2a). Moreover, the γH2AX MFI reductions of group BG + 2 Gy (32.85%) were significantly higher than those of group 2 Gy (18.49%) from 2 h to 24 h after IR (Figure 2a), which revealed a higher rate of DNA damage repair in group BG + 2 Gy and suggested that BG pretreatment enhanced the repair of DNA damage in EA.hy926 cells.

To assess the effects of BG pretreatment on cell apoptosis caused by carbon-ion irradiation, the cell apoptosis of each group was determined by flow cytometry based on annexin V-FITC/PI double staining. The results showed that the percentages of apoptosis in the 2 Gy and BG + 2 Gy groups were significantly higher than those of the control group (Figure 2b). Compared with group 2 Gy, the percentage of apoptosis in group BG + 2 Gy was significantly decreased at the same time point after IR (Figure 2b), revealing that BG pretreatment ameliorated the cell apoptosis caused by carbon-ion irradiation.

The cell cycle distribution was examined and the results are shown in Figure 2c. Carbon-ion irradiation caused serious S phase and G_2_/M phase arrest, as shown by significantly increased percentages of the S and G_2_/M phases in groups 2 Gy and BG + 2 Gy compared to the control. At 24 h after IR, the irradiated cells exhibited the most serious cell cycle arrests. BG pretreatment significantly decreased the G2/M phase arrests at 24 h and 36 h after IR, indicating that BG pretreatment could promote the recovery of cell cycle arrest caused by IR.

### 3.3. BG pretreatment modulated the redox status of EA.hy926 cells after carbon-ion irradiation

**Figure 3:**
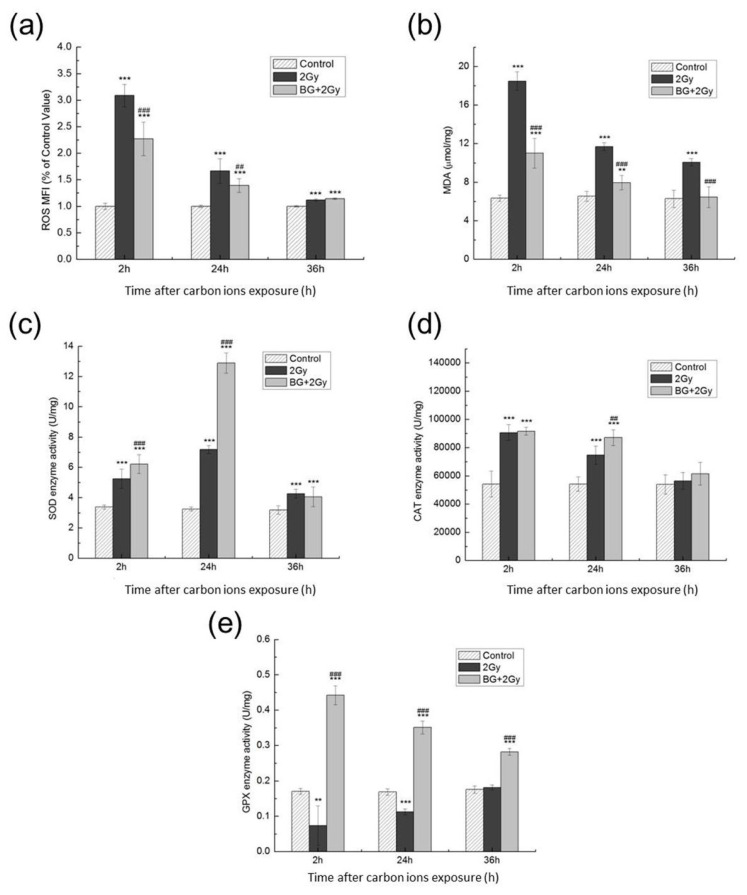
Effect of BG pretreatment on redox systems after 2 Gy carbon-ion irradiation. (a, b) ROS and MDA in cells were detected as hallmarks of oxidative stress. (c–e) SOD, CAT, and GPx enzyme activities in cells were measured to monitor antioxidant status. Data are presented as mean ± SE from three independent experiments. ^***^: p < 0.001, ^**^: p < 0.01 versus control group; ^###^: p < 0.001, ^##^: p < 0.01 versus IR group.

ROS and MDA can be used to quantitatively estimate oxidative lesions. To detect whether BG pretreatment could ameliorate carbon-ion irradiation-induced oxidative stress, we examined the ROS levels and MDA contents in each group of cells. As shown in Figures 3a and 3b, the ROS and MDA levels in the cells of groups 2 Gy and BG + 2 Gy were significantly increased in comparison to the control group at 2 h after IR and then gradually decreased with incubation time after IR. At 36 h after IR, the ROS and MDA in groups 2 Gy and BG + 2 Gy were still higher than those of the control group (p < 0.001), indicating that carbon-ion irradiation obviously triggered the production of ROS and oxidative stress. The ROS and MDA levels in group BG + 2 Gy were significantly decreased compared to those in group 2 Gy (p < 0.001) at the same time point after IR, indicating that pretreatment with BG significantly depressed the production of ROS and ameliorated the protein and lipid oxidation induced by carbon-ion irradiation.

The antioxidant status of each group is shown in Figures 3c–3e. The data revealed that the activities of SOD and CAT were increased in group 2 Gy (Figures 3c and 3d) as a response to IR-induced oxidative damage. However, the activity of GPx decreased at 2 h after IR and then gradually recovered over time (Figure 3e). Pretreatment with BG significantly elevated the activities of antioxidative enzymes including SOD (Figure 3c), CAT (Figure 3d), and GPx (Figure 3e). These findings suggested that BG ameliorated carbon-ion irradiation-induced oxidative damage, most likely by enhancing the antioxidative enzyme activities in EA.hy926 cells.

### 3.4. BG pretreatment activated Src and nuclear translocation of NF-κB and regulated its downstream target gene expression in carbon-ion irradiated cells

**Figure 4:**
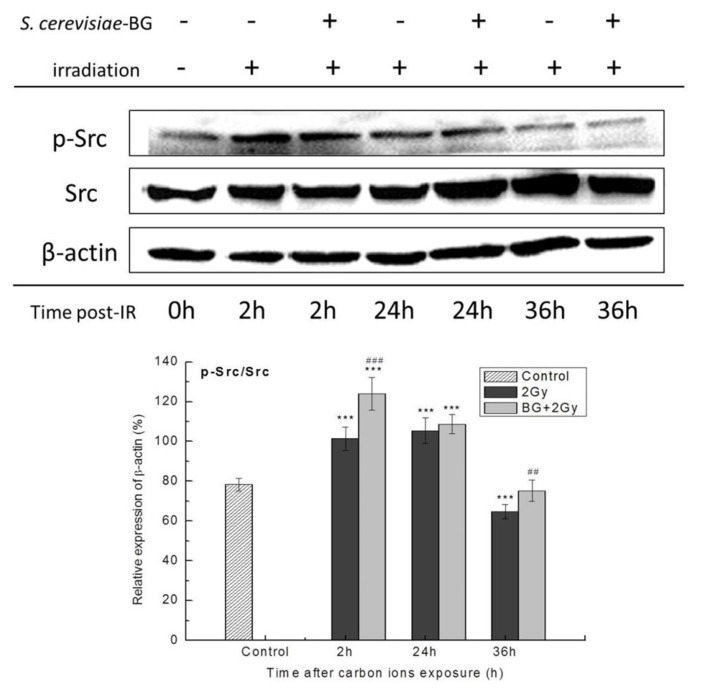
Effect of BG pretreatment on Src activation in carbon-ion irradiated cells. Western blotting analysis for p-Src (Tyr416) and Src with β-actin as loading control. Quantifications of average western blotting band intensities are represented as mean ± SE from three independent experiments. ^***^: p < 0.001 versus control group; ^###^: p < 0.001, ^##^: p < 0.01 versus IR group.

To explore the signaling triggered by the interaction of BG and dectin-1, the expression level of p-Src (Tyr416) was detected by western blotting assay. As shown in Figure 4, the expression of p-Src significantly increased at 2 h and 24 h after 2 Gy carbon-ion irradiation, and BG pretreatment further enhanced the p-Src expression, which indicated that Src was activated by irradiation and BG pretreatment intensified that activation.

**Figure 5:**
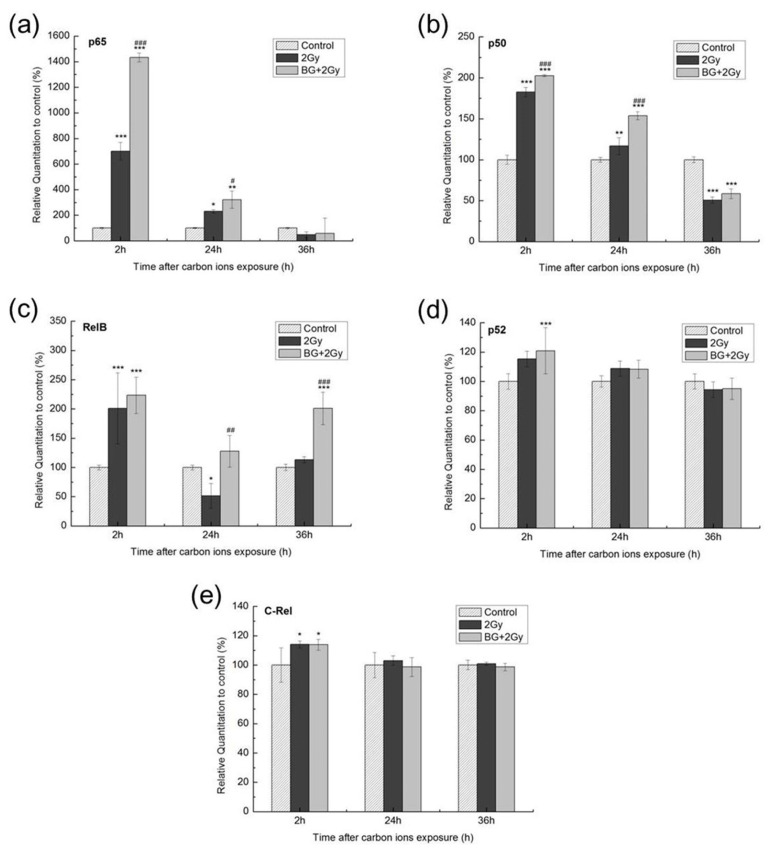
Effect of BG pretreatment on the binding activities of NF-κB after 2 Gy carbon-ion irradiation. (a–e) The abilities of activated NF-κB p65, p50, RelB, p52, and c-Rel subunits in binding to their consensus sequences were detected and relatively quantified, respectively. Data are presented as mean ± SE from three independent experiments. ^***^: p < 0.001, ^*^: p < 0.05 versus control group; ^###^: p < 0.001, ^##^: p < 0.01, ^#^: p < 0.05 versus IR group.

To assess the activation of NF-κB, we detected the binding activities of all of the NF-κB components, including p65, p50, RelB, p52, and c-Rel, using a transcription factor assay kit, and the results are shown in Figure 5. For p65 and p50, the activations at 2 h and 24 h following carbon-ion irradiation in group 2 Gy were higher than those of the control, and BG pretreatment further enhanced these activations (Figures 5a and 5b). For RelB, the activation at 2 h in group 2 Gy was higher than that of the control group, and BG pretreatment not only enhanced RelB activation at 2 h but also triggered RelB activation at 24 h and 36 h (Figure 5c). p52 was not activated by carbon-ion irradiation, but it was activated by BG at 2 h after carbon-ion irradiation (Figure 5d). For c-Rel, the activation levels at 2 h in groups 2 Gy and BG + 2 Gy were higher than those of the control group, and BG pretreatment did not enhance RelB activation (Figure 5e). These results indicated that carbon-ion irradiation mainly triggered the NF-κB p65 and p50 subunits, and the combination of BG and carbon-ion irradiation mainly increased the activation of the NF-κB p65 and p50 subunits and triggered the RelB subunit.

**Figure 6:**
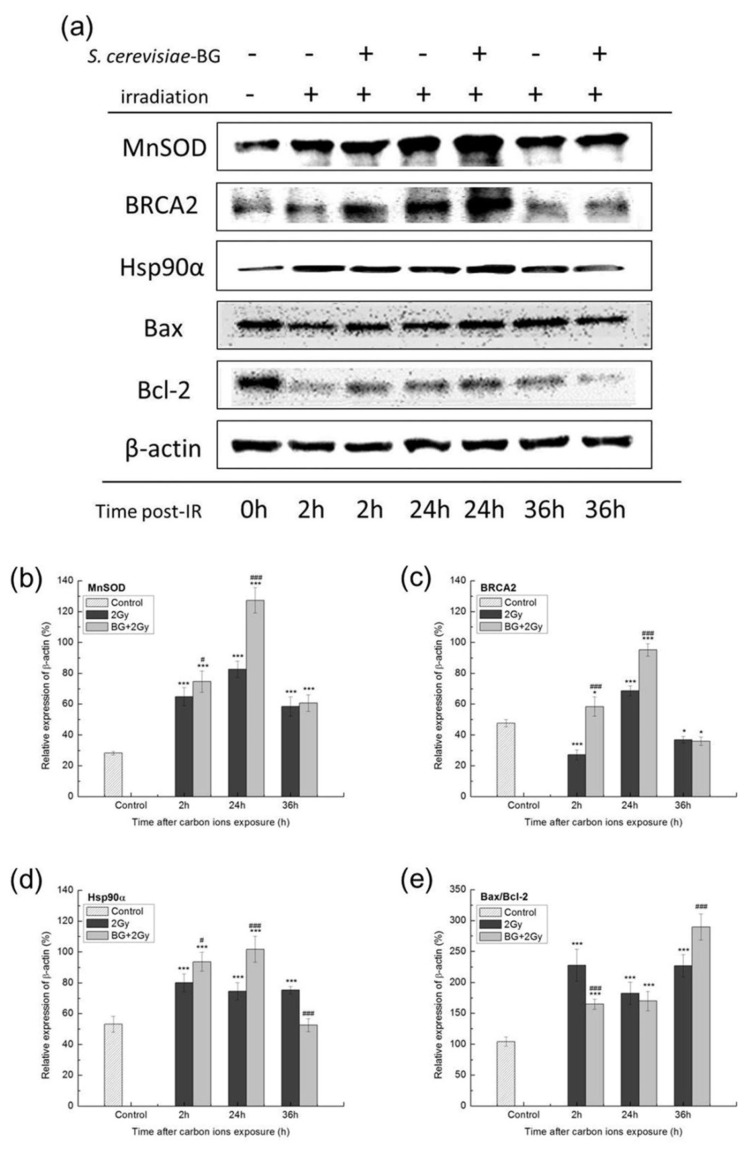
Effect of BG pretreatment on the protein expressions of target genes regulated by NF-κB. (a) Western blotting analysis for the indicated proteins with β-actin as loading control. (b–e) Quantifications of average western blotting band intensities are represented as mean ± SE from three independent experiments. ^***^: p < 0.001, ^*^: p < 0.05 versus control group; ^###^: p < 0.001, ^#^: p < 0.05 versus IR group.

NF-κB regulates the expression of diverse genes by binding to specific DNA elements, which contain the promoters/enhancers of the target genes. We investigated the protein expressions of several prosurvival-related genes directly regulated by activated NF-κB. These included antioxidant protein MnSOD, DNA damage-response and repair proteins BRCA2 and Hsp90α, and apoptosis regulatory proteins Bax and Bcl-2. The expression of these proteins was detected by western blotting assay and the results are shown in Figure 6.

The protein level of MnSOD in group 2 Gy was significantly increased at 2 h, 24 h, and 36 h after carbon-ion irradiation compared to the control group, and BG pretreatment further enhanced MnSOD protein expression (Figure 6b), suggesting that BG increased the ability of EA.hy926 cells to scavenge free radicals by increasing the expression of antioxidative enzymes.

The BRCA2 protein level in group 2 Gy was significantly increased at 24 h after carbon-ion irradiation compared to the control group. In group BG + 2 Gy, the BRCA2 protein levels at 2 h and 24 h were significantly higher than those in group 2 Gy and the control group (Figure 6c). The Hsp90α protein level in group 2 Gy was significantly increased at 2 h, 24 h, and 36 h after carbon-ion irradiation compared to the control group, and in group BG + 2 Gy, the Hsp90α protein levels at 2 h and 24 h were significantly higher than those of group 2 Gy (Figure 6d). These results showed that carbon-ion irradiation induced increased BRCA2 and Hsp90α expression levels to some degree. BG pretreatment enhanced irradiation-induced BRCA2 and Hsp90α protein expressions, indicating that BG could enhance the repair of carbon-ion irradiation-induced DNA damage by increasing BRCA2 and Hsp90α protein expression.

Apoptosis is modulated by various proapoptotic and antiapoptotic factors. Bax is a proapoptotic factor and Bcl-2 is an antiapoptotic factor, and the ratio of Bax to Bcl-2 is an important determinant of apoptotic cell death or survival. The data showed that carbon-ion irradiation increased the protein level of Bax and decreased the protein level of Bcl-2 (Figure 6a), and the ratio of Bax/Bcl-2 in group 2 Gy was significantly higher than that of the control (Figure 6e). BG pretreatment prevented the irradiation-induced increase in the Bax/Bcl-2 ratio (Figure 6e), suggesting that BG inhibited apoptosis and promoted cell survival by decreasing the Bax/Bcl-2 ratio.

## 4. Discussion

Studies have shown that β-glucan is a potential radioprotector [9], but most studies to date have focused on the protective effects of β-glucan on the immune and hematopoietic systems after X-/γ-ray irradiation [10,11]. In the present study, we chose the EA.hy926 human umbilical endothelial cell line as a model system to investigate the protective effect of BG against damage induced by high-LET carbon-ion irradiation and explored the possible mechanism of that action.

Based on our previous research [12], 10 μg/mL BG pretreatment for 24 h was used to study the protective effects of BG in EA.hy926 cells damaged by carbon-ion irradiation. It was found that BG pretreatment increased the clonogenic survival rate of cells exposed to different doses of carbon-ion irradiation (Figure 1), indicating that BG could protect EA.hy926 cells from carbon-ion radiation damage, thereby increasing the tolerance of EA.hy926 cells to carbon-ion irradiation. BG could also increase the tolerance of EA.hy926 cells [12] and other cells [18] to X-ray irradiation.

Most of the cytotoxicity induced by ionizing radiation is mediated by DNA DSBs. DNA DSBs are considered to be the main cause of radiation-induced cell death. Our results showed that 2 Gy carbon-ion irradiation caused serious DSBs, and unrepaired DSBs still significantly existed at 36 h after IR (Figure 2a). BG pretreatment decreased this DNA damage to a certain extent and significantly promoted the repair of DNA DSBs (Figure 2a). BG pretreatment before X-ray irradiation could also significantly decrease DNA damage [11,12]. Carbon-ion irradiation significantly induced cell apoptosis (Figure 2b). Cell apoptosis as a result of radiation is reduced by BG pretreatment, indicating the prosurvival role of BG for endothelial EA.hy926 cells (Figure 2b). In addition, BG pretreatment could also improve radiation-induced cell-cycle disorders (Figure 2c).

Ionizing radiation can perturb cellular redox homeostasis and induce oxidative stress via free radical generation. The increased ROS and MDA levels obtained in this study indicated that carbon-ion irradiation significantly perturbed cell redox homeostasis and shifted the redox balance of the cells toward oxidation, while BG pretreatment effectively decreased the levels of ROS and MDA (Figures 3a and 3b). Furthermore, BG significantly increased the SOD, CAT, and GPx enzyme activities (Figures 3c–3e), suggesting that BG increased the free radical scavenging ability by enhancing antioxidative enzymes activities in EA.hy926 cells. The elevated free radical scavenging ability could largely prevent the occurrence of radiation damage. BG pretreatment also significantly promoted DNA DSB repair (Figure 2a), implying that there are other ways for BG to stimulate cells to cope with existing damage.

The results of signal molecule analysis showed that the Src, NF-κB p65, and NF-κB p50 subunits were activated by carbon-ion irradiation, while the NF-κB RelB and NF-κB c-Rel subunits were also activated at 2 h after exposure (Figures 4, 5a–5c, and 5e). A similar molecular composition of ionizing radiation-activated NF-κB was also found in hairless mouse skin cells [19] and human monocyte leukemia cells [20], and downregulated NF-κB activation sensitized tissues such as the intestinal epithelium to ionizing radiation-induced damage [21]. These studies indicated that cells had heightened self-protection with the activation of the NF-κB signaling pathway in response to ionizing radiation-induced damage. BG pretreatment before carbon-ion irradiation activated the Src and NF-κB p65, p50, and RelB subunits, and their activation levels were significantly higher than those induced by irradiation alone (Figures 4 and 5a–5c). BG pretreatment also significantly activated the NF-κB p52 subunit at 2 h after carbon-ion irradiation (Figure 5d). These results indicated that the NF-κB signaling pathway in carbon-ion irradiated EA.hy926 cells was strongly triggered by *S. cerevisiae* BG. It was reported that the activation of Src triggered by lipopolysaccharides could induce NF-κB activation [22], suggesting that the interaction of BG with the dectin-1 receptor triggers Src activation and then induces NF-κB activation.

The activation of NF-κB regulates the expression of multiple genes by binding to specific DNA elements, which contain the promoters/enhancers of the target genes [23]. Based on the results of NF-κB activity (Figure 5), we concluded that for the expression levels of several proteins, including antioxidant protein MnSOD, DNA damage-response and repair-related proteins BRCA2 and Hsp90, and apoptosis regulatory proteins Bax and Bcl-2, their gene expressions were directly regulated by NF-κB.

MnSOD is a nuclear-encoded mitochondrial enzyme that protects cells from oxidative damage by converting superoxide radicals into hydrogen peroxide, which is further detoxified by catalase and glutathione peroxidases. MnSOD can be regulated by p65 and RelB because the κB binding sequences are found in its promoter [24,25]. Our results showed that BG pretreatment significantly increased the expression of MnSOD after irradiation (Figure 6b), which could further increase the MnSOD activity (Figure 3c) and suggested that BG increases the ability of EA.hy926 cells to scavenge free radicals by increasing the expression of NF-κB-regulated MnSOD. The elevated MnSOD protein levels (Figure 6b) and increased antioxidative enzyme activities (Figure 3) promoted the scavenging of free radicals and thus alleviated cell injury induced by carbon-ion irradiation. Some antioxidants such as resveratrol also reduced X-ray irradiation damage by increasing the mRNA expression of MnSOD [26]. Other studies reported that overexpression of MnSOD could protect tissues from X-/γ-ray irradiation [27,28].

BRCA2 is essential for efficient DNA homologous recombination repair, which is one of the most important pathways for repairing DSBs. The p65/p50 NF-κB heterodimer directly interacts with the NF-κB-like site in the BRCA2 promoter [29]. BG pretreatment significantly increased the BRCA2 protein level at 2 h and 24 h after irradiation while it was decreased at 36 h (Figure 6c), which was consistent with p65 and p50 activation (Figures 5a and 5b). Hsp90 is an evolutionarily conserved molecular chaperone. Hsp90 and its associated co-chaperones have important functions in the nucleus [30]. Hsp90 as an upstream regulator that participates in DNA repair [31]. Hsp90a is the major cytoplasmic isoform of Hsp90 [32]. Hsp90α can be regulated by NF-κB transcription factors through p65 binding to its promoter [33]. Our results showed that BG pretreatment clearly increased the expression of Hsp90α at 2 h and 24 h after irradiation. These results indicated that BG pretreatment promoted the repair of irradiation-induced DNA damage by increasing NF-κB-regulated BRCA2 and Hsp90a protein expressions. Both the BRCA2 and Hsp90 proteins play essential roles in DNA damage response and repair [34]. Knocking out the BRCA2-DNA repair complex increased the 5-fluorouracil-induced DNA damage in HCT-8 cells [35]. Inhibiting Hsp90 could sensitize tumor cells to carbon-ion irradiation [36,37]. These findings also suggested that increased BRCA2 and Hsp90 expressions may contribute to the repair of DNA damage.

Bax serves as a promoter of apoptosis and Bcl-2 acts as a repressor of apoptosis [38]. The ratio of Bax to Bcl-2 is an important determinant of apoptotic cell death or survival [39]. The p65/50 heterodimer can bind to the Bcl-2 promoter [40]. Bax protein levels were increased while Bcl-2 protein levels were decreased by 2 Gy irradiation, and so the Bax/Bcl-2 ratio increased after carbon-ion irradiation. BG pretreatment prevented the irradiation-induced increase in the Bax/Bcl-2 ratio at 2 h and 24 h after irradiation (Figure 6e). The time-dependent change in the Bax/Bcl-2 ratio (Figure 6e) was consistent with p65 and p50 activation (Figures 5a and 5b), indicating that *S. cerevisiae*-BG-induced NF-κB activation may contribute to decreased apoptosis. Flavonoids and melanin also prevented γ-ray irradiation-induced apoptosis by upregulating Bcl-2 expression [41] or decreasing the Bax/Bcl-2 ratio [42].

**Figure 7:**
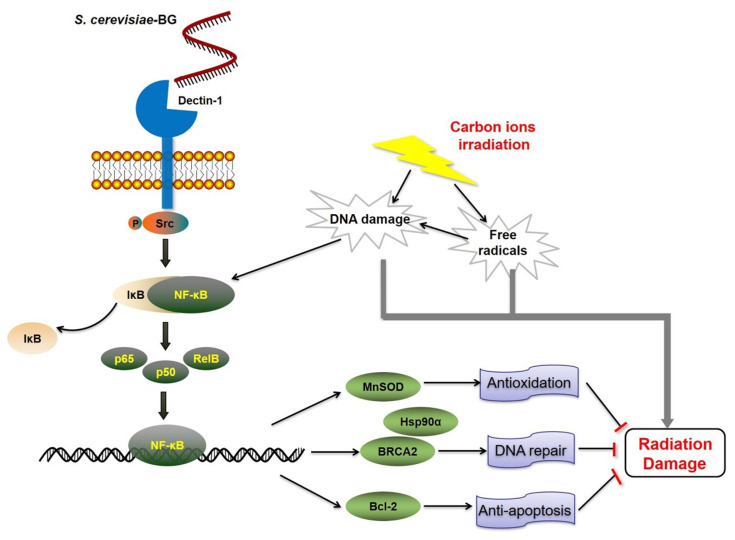
A schematic representation of *S. cerevisiae* β-glucan-induced signaling in carbon-ion irradiated human umbilical endothelial cells (EA.hy926).

Results at the molecular level indicated that Src was activated by the interaction of BG and the dectin-1 receptor. Phosphorylated Src activated p65, p50, and RelB, and the activated NF-κB upregulated the expression of antioxidant protein MnSOD, DNA damage-response and repair-related proteins BRCA2 and Hsp90α, and antiapoptotic protein Bcl-2. A schematic representation of BG-induced signaling through dectin-1/Src/NF-κB/target gene expression (MnSOD, BRCA2, Hsp90, and Bcl-2) in carbon-ion irradiated human umbilical endothelial cells (EA.hy926) is shown in Figure 7. Previous studies on the activation of NF-κB only involved some of the NF-κB components [43–45], but in the present study, the activations of all NF-κB components were evaluated to fully understand this activation. Most studies of polysaccharides have focused on their antioxidation and immunomodulatory abilities in the face of radiation damage [44,46,47]. Our study is the first to report that BG could upregulate prosurvival-related protein expression in irradiated cells.

In conclusion, the present study has shown that BG pretreatment ameliorates carbon-ion irradiation-induced EA.hy926 cell injury, enhances the repair of DNA damage, and increases clonogenic survival. The possible mechanism of BG exerting this radioprotective effect may be BG binding to the dectin-1 receptor and triggering Src activation, and then activating NF-κB, ultimately upregulating MnSOD, BRCA2, Hsp90α, and Bcl-2 gene expression. The elevated MnSOD level and antioxidant enzyme activities increase the ability to scavenge free radicals, the elevated BRCA2 and Hsp90a levels promote the repair of irradiation-induced DNA damage, and the elevated Bcl-2 levels decrease the Bax/Bcl-2 ratio and then decrease carbon-ion irradiation-induced apoptosis. Taken together, the upregulation of prosurvival-related gene expression and the increase of antioxidant enzyme activities mitigate EA.hy926 cell damage produced by high-LET carbon-ion irradiation and ultimately promote the survival of irradiated cells.
